# Is handgrip strength and key pinch measurement related with biochemical parameters of nutrition in peritoneal dialysis patients?

**DOI:** 10.12669/pjms.314.7595

**Published:** 2015

**Authors:** Bulent Yardimci, Abdullah Sumnu, Ibrahim Kaya, Meltem Gursu, Zeki Aydin, Serhat Karadag, Sami Uzun, Emel Tatli, Savas Ozturk, Ercan Cetinus, Rumeyza Kazancioglu

**Affiliations:** 1 Bulent Yardimci, Internal Medicine, Istanbul Florence Nightingale Hospital, Istanbul, Turkey; 2 Abdullah Sumnu, Nephrology, Haseki Training and Research Hospital, Istanbul, Turkey; 3 Ibrahim Kaya, Ortopedics and Traumatology, Haseki Training Hospital, Istanbul, Turkey; 4 Meltem Gursu, Nephrology, Haseki Training and Research Hospital, Istanbul, Turkey; 5 Zeki Aydin, Nephrology, Haseki Training and Research Hospital, Istanbul, Turkey; 6 Serhat Karadag, Nephrology, Haseki Training and Research Hospital, Istanbul, Turkey; 7 Sami Uzun, Nephrology, Haseki Training and Research Hospital, Istanbul, Turkey; 8 Emel Tatli, Nephrology, Haseki Training and Research Hospital, Istanbul, Turkey; 9 Savas Ozturk, Nephrology, Haseki Training and Research Hospital, Istanbul, Turkey; 10 Ercan Cetinus, Ortopedics and Traumatology, Haseki Training Hospital, Istanbul, Turkey; 11 Rumeyza Kazancioglu, Nephrology, Bezmialem Vakif University, Medical Faculty, Istanbul, Turkey

**Keywords:** Handgrip Strenngh, Key Pinch, Nutrition, Peritoneal Dialysis, Protein-energy wasting: PEW, End-stage renal disease: ESRD, Subjective global assessment: SGA, Handgrip strength test: HST, Peritoneal dialysis: PD, Hemoglobin: Hb, Thyroid stimulating hormone: TSH, Body Mass Index: BMI, Skinfold thicknesses: SFT, Standard deviation: SD, Analysis of variance: ANOVA.

## Abstract

**Backgrounds & Objective:**

End-stage renal disease (ESRD) frequently causes Protein Energy Wasting (PEW), which is an important morbidity and mortality factor. Although it is difficult to assess PEW with a reliable method, there are various methods such as Handgrip strength test (HST), serum albumin, cholesterol, etc. HST is a simple and reliable antropometric method which is used for nutritional status and body muscle strength. This study aims to assess the relationship between HST and biochemical markers in evolution of nutritional status of ESRD patients.

**Methods:**

This cross-sectional study included 36 consecutive patients, who are on peritoneal dialysis and 36 healthy –control subjects. Jamar-hand dynamometer was used for handgrip strength test; a pinch gauge was used for key pinch. Other antropometric tests included skin fold thicknesses at biceps, triceps, umbilical, suprailiac and subscapular regions; circumferences at waist hip, neck and midarm. Biochemical tests were performed only in Peritoneal Dialysis (PD) group. SPSS for Windows ver. 15.0 was used for statistics.

**Results:**

The mean age of patients was 49.3±14.4, and mean age of control group was 43.8±10.6 (p=0.075). In PD group dominant hand dynamometer test 1,2 and 3 results were 19.3±9.3 kg, 25.3±10.8 kg, 25.5± 10.6 kg and; 34.2±10.3 kg, 34.4±9.8 kg, 34.6±10.0 kg for control group (p< 0,001). Right key pinch results were 6.7±1.9 kg for patients; 13.5±4.5 kg for control group (p<0.001). Left key pinch results were 6.8±1.9 kg for patients; 13.2±4.4 kg for control group (p<0.001). There was not any significant relationship concerning handgrip or key pinch tests with biochemical parameters.

**Conclusion:**

Handgrip Strength Test and key pinch may be reliable, cheap and easily performed tests for the diagnosis of Protein Energy Wasting in patients on Peritoneal Dialysis.

## INTRODUCTION

Protein-energy wasting (PEW) is common in patients with end-stage renal disease (ESRD) and is a strong predictor of morbidity and mortality in dialysis patients. Although the factors contributing to PEW include decreased energy or protein intake, catabolic stimulus of hemodialysis itself, loss of nutrients and amino acids into dialysate, metabolic acidosis, resistance to insulin and anabolic hormones, such as growth hormone, the most important factor is inflammation.^1^-^5^ There is no reliable method to establish the diagnosis of PEW in dialyzed patients. To determine the nutritional status in dialysis patients; several biochemical, nutritional and clinical parameters such as levels of serum albumin, transthyretin, cholesterol, dietary protein intake, subjective global assessment (SGA), handgrip strength test (HST), muscle and fat mass reserve by use of anthropometry or bioelectrical impedance have been used. The most frequently used one among these parameters is serum albumin. However, there are many other factors, which affect the levels of albumin. These include negative acute phase response, peritoneal albumin loss in peritoneal dialysis (PD) patients, hemodilution by fluid overload, systemic diseases and old age.^6^,^7^

Muscle wasting is a good marker of PEW in ESRD patients.^8^,^9^ HST is a simple, inexpensive and easily performed bedside test, among the methods, which asses muscle reserves. Although it is not included in diagnostic criteria of PEW by International Society of Renal Nutrition and Metabolism^10^, HGS has been used as a nutritional marker both in hemodialysis and peritoneal dialysis patients.^1^,^11^ It is also to reflect the lean body mass and predict survival in PD patients.^8^,^11^ Nevertheless there is still a lack of evidence concerning the relationship of biochemical parameters and HST. In the present study, our aim was to determine the relationship between biochemical parameters of nutrition with HST and key pinch power in patients on PD.

## METHODS

In this cross-sectional study, consecutive patients on PD for at least 3 months at Haseki Training hospital were included. Patients age less than 18 or more than 80, those with dialysis duration of less than 3 months, active infectious or inflammatory disease within the last 3 months, any upper limb malformation and those who do not meet the dialysis adequacy criteria, and who had a functioning av fistula were excluded from the study. Adequacy of PD was determined by measuring weekly Kt/V urea and creatinine clearance by standard peritoneal equilibration test. All study participants provided signed informed consent at time of study enrollment.

The age, gender, primary kidney disease, co-morbidities and PD duration of all patients were recorded. Fasting venous blood samples were collected for the measurement of serum creatinine, albumin, phosphorus, total cholesterol, LDL-cholesterol, HDL-cholesterol, hemoglobin (Hb), HbA1c and thyroid stimulating hormone (TSH). The control group was consisted of healthy subjects in age-and-sex matched fashion.

Anthropometric measurements were performed at the outpatient clinics by the same physician with the patient wearing usual daily clothes but no shoes. Among these weight, height and body mass index (BMI=weight/height^2^) were recorded. Circumferences at waist, hip, neck and midarm were measured using tape measure. Skinfold thicknesses (SFT) at biceps, triceps, umbilical, suprailiac and subscapular region were measured by skin fold caliper. Every measurement was repeated for three times and the average value was recorded.

A calibrated Jamar dynamometer (Smith and Nephew, Irwington, NY 10533, USA) having 5 settings, was used to assess HST at the dominant arm. The American Society of Hand Therapists’ recommendations for testing both grip and pinch strengths were followed.^12^ Patients were seated comfortably on a chair without armrests. The shoulder was adducted, the elbow flexed at 90°, with the forearm and wrist in neutral position. After a warm-up section, patients were instructed to squeeze the handgrip as hard as they could. Patients were directed by the physician with the same tone of voice. The first 3 settings of dynamometer were used. Three trials for each setting were performed with a rest period at least one minute between the settings. A pinch gauge (PG-30, B&L Engineering Santa Fe, CA, USA) was used to assess the key pinch. Three measurements were repeated like HST and the highest score was recorded in kilograms.

Statistical analysis was carried out by using SPSS for Windows ver. 15.0. Quantitative data were given as mean value ± standard deviation (SD). Correlation analysis was performed by using Pearson analysis for numeric variables and Spearman’s Rho correlation test for nonnumeric variables. Pearson chi-square test and Fisher’s exact test were used for comparison between categorical variables. Baseline numeric parameters of the groups were compared with analysis of variance (ANOVA), if the distribution of parameter was normal, or with Kruscal Wallis-H test, if the distribution of parameter was abnormal. Tukey HSD was used in one-way ANOVA for post-hoc comparison whereas Mann Whitney U test was used for Kruscal Wallis-H; it was evaluated by applying Bonferroni correction to alpha significance level. p<0.05 was accepted as statistically significant.

## RESULTS

Thirty-six patients (18 male, 18 female) and 36 control (18 male 18 female) were included in the study. The mean age of the patients was 49.3±14.4 years and the mean time on PD was 44.8±26.9 months. The mean age of control group was 43.9±10 years (p=0.075). Primary kidney disease was diabetic nephropathy in 11 (31%), nephrosclerosis in 4(11%), autosomal dominant polycystic kidney disease in 4 (11%), chronic pyelonephritis in 3 (8%), urologic pathologies in 3 (8%), amyloidosis in 2 (6%) and chronic glomerulonephritis in 2 (6%) patients; while it was unknown in 7 (19%) patients.

All patients had reached optimal dialysis adequacy criteria with the weekly Kt/V, residual creatinine clearance and dialysate creatinine clearance values as 2.52±0.68; 34.48±33.18 ml/week/1.73m^2^ and 45.09±12.01ml/week/1.73m^2^; respectively. The mean daily urine output was 883±707 ml/day while seven patients had urine volume less than 200 ml/day. The mean proteinuria was calculated as 648±58.6 mg/day. The results of biochemical analysis are presented in Table-I.

**Table-I:** The results of biochemical and hematological analysis.

	Mean ± SD
Urea (mg/dl)	102,64±28,20
Creatinine (mg/dl)	8,19±2,91
Ca(mg/dl)	9.21±0.72
P(mg/dl)	4.89±1.48
Parathormone (pg/ml)	522±45.6
Total protein (g/dl)	6.67±0.81
Albumin (g/dl)	3.68±0.40
TSH (microIU/ml)	5.08±1.68
Total cholesterol (mg/dl)	193±45
LDL-cholesterol (mg/dl)	113±36
HDL-cholesterol (mg/dl)	42±16
Triglyceride(mg/dl)	177±14.1
Hemoglobin (g/dl)	11.06±1.57
Hematocrit (%)	33.21±4.73
CRP (mg/L)	1.58±2.16
HbA1c (%)	6.49±1.28

The right arm was the dominant arm in all patients. The results of anthropometric measurements are presented in Table-II. The handgrip strengths at levels 1, 2 and 3 on the dominant right hand were 19.29±9.38 kg, 25.30±10.85 kg and 23.50±10.68 kg respectively. Key pinch measurements on the right and left sides were 6.75±1.93 kg and 6.85±1.96 kg, respectively.

**Table-II:** Results of anthropometric measurements of both groups.

	Patient’s Mean + SD	Control Mean + SD	P
Age	49.30 ± 14.44	43.88±10.68	0.075
BMI (kg/m^2^)	27.75 ± 6.70	28.33 ± 6.16	0.720
Waist Circumference (cm)	96.94 ± 14.99	96.16 ± 18.87	0.850
Hip Circumference (cm)	99.41 ± 9.26	105.44 ± 8.75	0.007
Neck Circumference (cm)	36.95 ± 3.88	38.37 ± 5.42	0.215
Midarm Circumference (cm)	29.71 ± 4.64	30.04 ± 3.66	0.747
Biceps SFT (cm)	9.67 ± 5.66	12.11 ± 5.18	0.065
Triceps SFT (cm)	16.95 ± 9.94	18.50 ± 6.15	0.435
Suprailiac SFT (cm)	21.58 ± 14.52	20.40 ± 7.62	0.680
Umblical SFT (cm)	21.05 ± 13.02	30.80 ± 9.65	0.001
Subscapular SFT (cm)	22.70 ± 12.21	23.55 ± 9.60	0.747
Right Jamar 1 (kg)	19.29 ± 9.38	34.19 ± 10.36	<0.001
Right Jamar 2 (kg)	25.30 ± 10.85	34.41 ± 9.79	<0.001
Right Jamar 3 (kg)	23.50 ± 10.68	34.63 ± 10.02	<0.001
Left Jamar 1 (kg)	18.24 ± 8.08	32.77 ± 10.43	<0.001
Left Jamar 2 (kg)	23.86 ± 10.06	33.27 ± 10.42	<0.001
Left Jamar 3 (kg)	22.41 ± 9.68	33.08 ± 10.33	<0.001
Right Pinch (kg)	6.74 ± 1.93	13.58 ± 4.50	<0.001
Left Pinch (kg)	6.84 ± 1.96	13.27 ± 4.44	<0.001

Males were found to have higher values for Jamar-1 (23.00±10.82 kg vs. 15.36±5.53 kg; p=0.014), Jamar-2 (30.41±12.26 kg vs. 19.89±5.46 kg; p=0.003), Jamar-3 (28.77±11.68 kg vs. 17.93±5.77 kg; p=0.002), neck circumference (39.47±3.56 cm vs. 34.72±2.61 cm; p<0.001), urea (114±26 mg/dl vs. 90±24 mg/dl; p=0.008), creatinine (9.15±3.47 mg/dl vs. 7.17±1.76 mg/dl; p=0.042); and lower values for biceps SFT (7.62±4.03 cm vs. 11.5±6.36 cm; p=0.045), total cholesterol (166±35 mg/dl vs. 221±37 mg/dl; p<0.001), LDL-cholesterol (95±30 mg/dl vs. 132±32 mg/dl; p=0.002), HDL-cholesterol (39±9 mg/dl vs. 49±19 mg/dl; p=0.014), Kt/V (2.17±0.52 vs. 2.85±0.66; p=0.002) and residual urine volume (654±55.8 ml/day vs. 1112±81.2 ml/day: (p=0.046).

The presence or absence of neuropathy did not differ Jamar values while patients with neuropathy due to any reason (n=6) had lower values for right pinch (5.15±3.18 kg vs. 7.31±1.40 kg; p=0.017) and left pinch (5.33±2.35 kg vs. 7.20±1.67 kg; p=0.036).

HST findings were no different in patients with or without diabetes mellitus; while diabetic patients (n=11) had higher Hemoglobin (11.84±1.35 g/dl vs. 10.70±1.56 g/dl; p=0.043), subscapular SFT (30.6±10.6 cm vs. 19.4±11.5 cm; p=0.013), umbilical SFT (29.0±12.7 cm vs. 17.7±11.9 cm; p=0.019); lower calcium levels (8.86±0.78mg/dl vs. 9.37±0.64 mg/dl; p=0.049) and shorter dialysis duration (30±25 months vs. 51±25 months; p=0.026).

The results of statistically significant correlation analysis related to HST values are presented in Table-III. The determinants of handgrip and key pinch measurements in multivariate analysis are presented in Table-IV.

**Table-III:** The results of statistically significant correlation analysis related to HST values.

		R	P
Jamar-1	Right pinch	0.67	<0.001
	Left pinch	0.73	<0.001
	Albumin	0.34	0.047
	Total protein	0.44	0.01
	Neck circumference	0.41	0.018
	HbA1c	-0.61	0.003
Jamar-2	Right pinch	0.77	<0.001
	Left pinch	0.85	<0.001
	Height	0.42	0.014
	Neck circumference	0.47	0.006
	HbA1c	-0.55	0.008
	HDL cholesterol	-0.37	0.029
	Weight	0.43	0.012
	Total protein	0.50	0.003
Jamar-3	Height	0.50	0.003
	Weight	0.47	0.005
	Neck circumference	0.50	0.003
	CRP	0.34	0.046
	HbA1c	-0.50	0.017
	HDL cholesterol	-0.38	0.023
	Midarm circumference	0.36	0.037
	Total protein	0.45	0.009
	Right pinch	0.80	<0.001
	Left pinch	0.87	<0.001
Right pinch	Midarm circumference	0.35	0.047
	Total protein	0.53	0.002
Left pinch	HbA1c	-0.48	0.024
	Weight	0.38	0.032
	Total protein	0.46	0.009

**Table-IV:** Results of linear regression analysis of determinants of HST parameters.

	Determinants
Jamar-1	Left pinch, HbA1c.
Jamar-2	Left pinch.
Jamar-3	Gender, left pinch, right pinch, left pinch, height, weight, neck circumference, midarm circumference, CRP, HbA1c, HDL-cholesterol, total protein.
Right pinch	Jamar-3, presence of neuropathy.
Left pinch	Jamar-3, presence of neuropathy.

## DISCUSSION

Biochemical analysis is a well known assessment tool for nutritional status of the patients. However there are many factors, which interfere with them. Therefore further parameters including dietary protein intake, SGA, anthropometry and HST are needed for practical use in patients, who are on PD therapy.^3^,^11^ In the present cross-sectional study, we evaluated the association between hand grip strengths and key pinches with biochemical parameters of nutrition in patients on PD.

Handgrip strength has been shown to be a reliable indicator of skeletal muscle function in general population.^13^ But there are no clear cut values representing muscle wasting and yet there is no consensus about the technique and the extremity to be used.^14^,^15^ For this reason, we used control group. We found lower HST and key pinch values in PD group (Table-II). Theoretically, HST may be reduced in dialysis patients due to many factors such as water and electrolyte disturbances, hypotension episodes, hyperparathyroidism, dialysis related amyloidosis, carnitine deficiency and inadequate dialysis. However HST is related with hemoglobin levels, presence of anemia and decreased residual renal function; but not with dialysis adequacy.^11^-^16^

There have been several studies regarding the use of HST as a marker of nutritional status levels. In the study by Jones et al.^17^, there was no association between serum albumin and HST in PD patients. HST was found to be lower in patients with malnutrition assessed by SGA.^1^,^4^ Nevertheless Wang et al has shown that HST is correlated to serum albumin, hemoglobin levels and lean body mass; but there was no correlation with C-reactive protein.^11^ In this prospective study, HST was independently associated with all-cause and cardiovascular mortality in PD patients, regardless of serum albumin.^11^ Recently, Leal et al.^15^ used HST to assess the nutritional status of patients on Hemodialysis (HD) patients using a population-based set of reference values. In contrast to Wang et al.^11^, showed no correlation between HST and biochemical (serum albumin and hemoglobin) parameters.^16^ We found correlation between HST and serum protein, CRP and weight which, are nutrition parameters (Table-IV).

Many factors affect the hand grip and pinch strength like age, gender, BMI, hand dominance^11^,^14^, occupation^18^, and the technique used during the measurement.^19^ Our data showed that gender, height, neck circumference, midarm circumference and presence of neuropathy were related with HST as seen at Table-IV.

In the present study, although it has certain limitations as the sample size was rather small and it was designed as a cross-sectional study, we did not detect any significant correlation with biochemical parameters and handgrip measurements. Nevertheless, handgrip strength and key pinch can be used as a reliable marker of nutrition since it is a cheap test and it is easy to perform.
